# The socioeconomic impact of the chikungunya viral epidemic in India

**Published:** 2007-10-22

**Authors:** C Jairaj Kumar, C Arvind Baboo, B Unni Krishnan, Arunachalam Kumar, Shevonne Joy, Tom Jose, Anusha Philip, Kuraparthy Sambasivaiah, Belle Monappa Hegde

Infectious diseases in the developing and underdeveloped world are prime factors responsible for poor health status and poverty.^1^, ^2^ The recent chikungunya viral epidemic in India highlights the impact of a debilitating infection in working populations and its particular impact on those from low socioeconomic backgrounds. Chikungunya virus is a mosquito-transmitted alphavirus belonging to the family Togaviridae, and is endemic in Africa, India and Southeast Asia. It causes an acute infection of abrupt onset, characterized by high fever, arthralgia, myalgia, rash, photophobia and retro-orbital pain. Symptoms generally last about one week and recovery is usually complete.^3^

Almost two million cases were reported in India between February and August 2006.^3^ More cases have been reported in 2007.^4^, ^5^ Despite the high prevalence of chikungunya infection there are no reports on the impact of poverty and socioeconomic profile on the spread of the disease or morbidity experienced.

We sought to determine the relation between poverty and infection using a cross-sectional, hospital-based study of 3 541 consenting patients from three states in South India with clinically confirmed chikungunya during the epidemic from February to August 2006 (see Appendix for clinical criteria). We present data on demographic and socioeconomic characteristics of the patients and on their period of morbidity.

Our findings reveal that 80% (2 832/3 541) of chikungunya-affected patients were below the poverty line according to the World Bank’s definition of income level less than $1US per person per day (the calculated average family size was 4.5). Almost two-thirds (64%, 2250/3541) of infections occurred in the most productive age group of 15–45 years (mean 32; Table 1), and many (62%, 2 189/3 541) patients experienced morbidity related to their infection for more than 15 days. One-quarter (27.5%) suffered for more than 1 month (Table 2). High-income participants (monthly household income > US$225) reported a significantly longer morbidity period, especially among those whose symptoms lasted longer than 30 days (*p* ≶ 0.0001)(Table 2). Infection was significantly more common in lower income groups across all age groups (*p* < 0.0001).

Anecdotal evidence from our data collection suggests that families of patients such as farm labourers or those working for a daily wage were often deprived of meals because of reduced income. Children also suffered when affected mothers were unable to care for their daily nutritional requirements. Problems in achieving adequate nutrition also seemed to be exacerbated by many participants contracting simultaneous diarrheal infections.

**Table 1:**
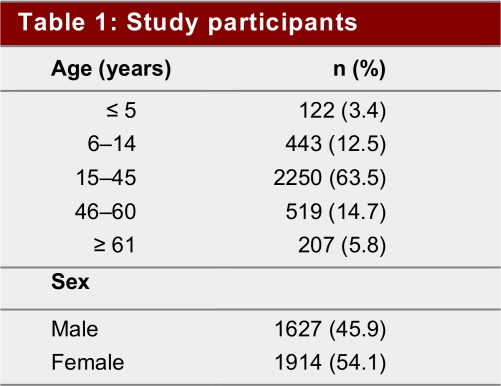
Study participants

**Table 2:**
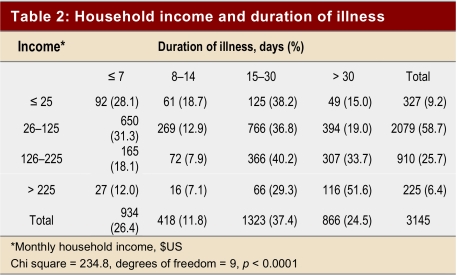
Household income and duration of illness

Our data suggest that poverty is an important determinant of chikungunya infection, and, further, that chikungunya infection exacerbates the problems of poverty. Poor people are most commonly affected, and as a high proportion are in the productive working age group, with symptoms in many lasting for over 2 weeks, many suffer a lack of income as a result. It is possible that the observed link between poverty and infection can be partly explained by current research showing that malnutrition enhances individual susceptibility to infectious disease by lowering immunity.^6^

If this is the case, possible government interventions include breaking the cycle of malnourishment, infection and further malnourishment by providing therapeutic food formulations to poor individuals. Funds for these kinds of initiatives are available, given that half the central government funds allocated to fight chikungunya were unspent last year.^7^ That being said, we are concerned that state government plans to initiate low-cost health coverage plans for those below the poverty line may be delayed because of the epidemic.^7^

The chikungunya epidemic also highlights the centrality of governmental preventive measures for vector-borne diseases, such as clearing vector breeding places, providing health education on preventing mosquito bites, and so forth. Government support for research on infection and malnutrition is also clearly warranted for scientific, economic and ethical reasons.^5^

The impetus to control chikungunya infection goes beyond the individual: tourism to chikungunya-affected regions is depressed,^8^ and there is evidence of population migration with attendant problems of overcrowding and poor housing.^9^ These could also exacerbate problems of poverty if employment requirements are altered, e.g., for tourism-related activities.

Interestingly, a longer duration of illness is reported in high-income participants, especially for those experiencing morbidity greater than 30 days. We propose that this may be because they can afford to take rest until complete recovery, while manual labourers or those working for daily wages cannot afford to do so. Further examination of this and the relation between infection and nutritional status is warranted.

Our results should be interpreted with caution. Although we believe that patients attending the hospitals surveyed are representative of the general population, it is possible that wealthier patients attend private clinics. We believe this is unlikely, however, given that the participating hospitals had expert clinics for patients with chikungunya infections and that the participating government hospitals have special wards for high-income patients and are teaching hospitals attached to highly respected medical colleges in India. A further limitation of our study is that we relied on a clinical diagnosis of chikungunya in the absence of a diagnostic test.

Although it is largely believed that complications of chikungunya are not serious, illness in individuals from poor backgrounds can have serious consequences, such as reduced productivity at the individual and community level, malnutrition, other infections, socioeconomic instability and exacerbation of poverty. Attention to the links between poverty, illness and human development are key in future research and program development related to chikungunya infection.

## Figures and Tables

**Figure:**
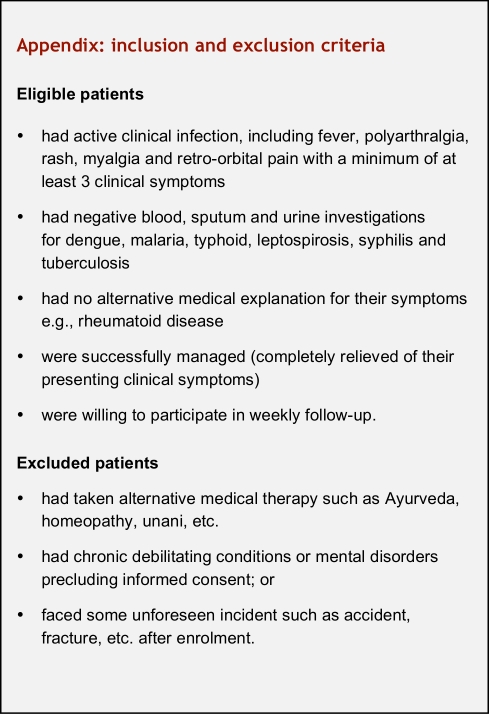
Inclusion and exclusion criteria
